# Influenza A-Associated Acute Necrotizing Encephalitis With Complete Recovery Following Early Immunomodulatory Therapy

**DOI:** 10.7759/cureus.103739

**Published:** 2026-02-16

**Authors:** Adeola A Adeleke, Sarah M Wall, Shannon L Andrews, Beth K Thielen

**Affiliations:** 1 Primary Care, Touro College of Osteopathic Medicine, Middletown, USA; 2 Pediatric Infectious Diseases, University of Minnesota School of Medicine, Minneapolis, USA

**Keywords:** acute necrotizing encephalopathy, cytokine dysregulation, immunomodulatory therapy, influenza-associated encephalopathy, pediatric influenza a

## Abstract

We describe a case of a previously healthy 19-month-old female who experienced rapid neurological deterioration following infection with influenza A (H1N1) and was found to have neuroimaging features characteristic of acute necrotizing encephalitis (ANE), including bilateral thalamic involvement and additional multifocal, symmetric gray- and white-matter lesions. At initial presentation, multiple proinflammatory cytokines were markedly elevated in the peripheral blood. Interestingly, while multiple cytokines were also elevated in cerebrospinal fluid (CSF), interleukin-6 (IL-6) and IL-8 levels were substantially higher in CSF (590 and 1330 pg/ml, respectively) than in serum (118 and 68.2 pg/ml, respectively), providing insights into disease pathogenesis and central nervous system-specific immune activation. The patient was treated in a stepwise manner with multimodal immunomodulatory therapy, including intravenous immunoglobulin (days 1-2), corticosteroids (start day 1), IL-1 receptor antagonist anakinra (start day 2), and plasma exchange (start day 3). Blood cytokine levels declined rapidly with initiation of immunomodulatory therapy. She demonstrated a favorable clinical response with complete neurological recovery, despite an unfavorable ANE severity score of 6 that has historically been associated with high mortality. This case highlights the importance of early recognition of ANE in young children with recent influenza infection, the need for prompt multidisciplinary management, consideration of targeted immunomodulatory therapy, and further investigation of cytokine profiling as a tool to improve diagnosis and guide treatment.

## Introduction

Influenza is among the most common respiratory viral infections worldwide, causing annual epidemics with significant morbidity and mortality. While most cases are self-limited, influenza can give rise to severe complications, particularly in young children, older adults, pregnant individuals, and those with underlying medical conditions or immunocompromising states. Beyond primary viral pneumonia and secondary bacterial superinfections [1,2], influenza has been implicated in a wide spectrum of systemic manifestations [3]. Although less frequently recognized, influenza can also affect the central nervous system. Reported neurological complications include febrile seizures, influenza-associated encephalopathy (IAE), acute cerebellar ataxia, transverse myelitis, Guillain-Barré syndrome, and postinfectious encephalitis (also known as acute disseminated encephalomyelitis). These manifestations may arise from direct viral invasion, post-infectious immune-mediated mechanisms, or systemic effects such as hypoxia and metabolic derangements [3]. Their severity varies widely, ranging from transient confusion to life-threatening cerebral injury with lasting neurological deficits.

In January 2025, the Centers for Disease Control and Prevention (CDC) received reports of nine deaths in children due to severe IAE [4] and requested clinician and health department reporting of similar cases on February 28, 2025 [5]. Here we describe a case identified in the setting of heightened awareness following this alert.

## Case presentation

A 19-month-old previously healthy girl from Minnesota presented with acute neurological deterioration following a recent influenza infection. She was up to date on routine childhood immunizations with the exception of influenza and COVID-19 vaccines. Several days prior to admission, the patient developed respiratory symptoms concurrent with multiple family members, all of whom tested positive for influenza. She did not receive any antiviral treatment.

On the day prior to admission, she developed a new fever and associated fatigue. On the morning of admission, the patient experienced a witnessed seizure characterized by stiffening, arching movements, and subsequent unresponsiveness. She was transported by private vehicle to a nearby adult emergency department, where her initial examination revealed a Glasgow Coma Scale (GCS) score of 3 with ongoing seizure-like activity, and she was ultimately determined to have been in status epilepticus for at least an hour prior to arrival. On exam, she was hypoxemic (O2 saturation 84%), febrile to 108 °F (rectal), and exhibited eyelid fluttering and upper extremity stiffening. She was intubated for airway protection, external cooling was initiated, and she received a loading dose of levetiracetam for seizure control, with gradual normalization of her body temperature. Blood and urine cultures were obtained, and appropriate empiric therapy with ceftriaxone and vancomycin was initiated for possible bacterial meningitis. Given concern for herpes simplex virus (HSV) encephalitis, a blood HSV polymerase chain reaction (PCR) was sent and was ultimately negative, and empiric acyclovir was started prior to transfer to the pediatric intensive care unit (PICU) until the test returned. 

Hospital course

In the PICU, she was hypotensive with signs of poor perfusion on admission despite 60 ml/kg of crystalloid fluid boluses and required epinephrine infusion to support blood pressure. Initial laboratory studies (Table 1) were notable for lactic acidosis, mild elevations in liver enzymes, and an elevated procalcitonin level in the setting of normal C-reactive protein (CRP) and erythrocyte sedimentation rate (ESR). A lumbar puncture revealed clear and colorless cerebrospinal fluid (CSF) with elevated protein, glucose, and red blood cells; normal white blood cells, and a negative meningitis/encephalitis panel (BioFire, USA). Repeat nasopharyngeal swab testing for respiratory pathogens was positive for influenza A (H1N1 subtype), so oseltamivir 30 mg BID was initiated.

**Table 1:** Serial laboratory values during hospitalization (days 1–6) Laboratory results with reference ranges for comprehensive metabolic panels, complete blood counts, coagulation studies, inflammatory markers, and cerebrospinal fluid (CSF) studies during the first six days of hospitalization. Abbreviations: ALT, alanine aminotransferase; AST, aspartate aminotransferase; INR, international normalized ratio; PTT, partial thromboplastin time; CRP, C-reactive protein; ESR, erythrocyte sedimentation rate

Laboratory values	Reference range	Hospital day					
		1	2	3	4	5	6
Comprehensive metabolic panel							
Sodium (mmol/L)	135-145	140	144	139	140	141	141
Potassium (mmol/L)	3.4-5.3	4.4	2.8	3.4	3.5	3.5	3.2
Chloride (mmol/L)	98-107	102	113	109	107	106	101
Carbon dioxide (mmol/L)	22-29	20	21	19	24	29	29
Urea nitrogen (mg/dL)	5.0 - 18.0	25.8	21	13.6	11.5	9.9	12.7
Creatinine (mg/dL)	0.18 - 0.35	0.68	0.41	0.39	0.28	0.23	0.22
Calcium (mg/dL)	9.0 - 11.0	9.5	8.7	7.8	7.9	7.9	8.5
Magnesium (mg/dL)	1.6 - 2.7	2.3	-	1.8	1.7	1.5	-
Phosphorus (mg/dL)	3.4 - 6.0	5.2		2.7	1.9	3	2.8
Anion gap (mmol/L)	7 - 15	18	11	11	9	9	11
Lactic acid (mmol/L)	0.7 - 2.0	4.2	0.7	0.7	2.7	2.9	2.7
Liver studies							
ALT (U/L)	0 - 50	32	82	52	32	33	-
AST (U/L)	0 - 60	157	163	70	33	29	-
Alkaline phosphatase (U/L)	110 - 320	173	128	101	76	63	-
Total bilirubin (mg/dL)	<1.0	0.2	<0.2	<0.2	0.2	0.2	-
Albumin (g/dL)	3.8 - 5.4	3.9	3.1	2.8	3.2	3.7	-
Total protein (g/dL)	5.9 - 7.3	7	6.3	7	5.8	5.6	-
Ammonia (umol/L)	11-51	-	19	18	-	-	-
Complete blood count							
White blood cell count (10^3^/µL)	6.0 - 17.5	9.8	3.7	2.7	1.9	1.9	3
Absolute neutrophil count (10^3^/µL)	0.8 - 7.7	3.9	2.8	1.6	1.2	1.3	2.2
Absolute lymphocyte count (10^3^/µL)	2.3 - 13.3	5.1	0.6	1.1	0.6	0.4	0.6
Hemoglobin (g/dL)	10.5 - 14.0	13.3	10.6	9.6	10.8	10.7	10.1
Platelet count (10^3^/µL)	150 - 450	256	88	63	66	59	134
Coagulation							
INR	0.85 - 1.15	-	1.72	1.26	1	0.94	1.02
PTT (s)	22 - 38	-	49	38	33	29	28
Fibrinogen (mg/dL)	170 - 510	-	222	180	187	176	180
Inflammatory markers							
CRP (mg/L)	<5.00	<3.0	23.84	4.96	<3.0	<3.0	<3.0
ESR (mm/hr)	0 - 15	11	-	-	-	-	-
Procalcitonin (ng/mL)	<0.50	98.27	-	-	-	-	-
Ferritin (ng/mL)	8 - 115	-	3,692	1,293	310	183	175
CSF studies							
Protein (mg/dl)	15.0 - 45.0	121.3	-	-	-	-	-
Glucose (mg/dL)	40 - 70	74	-	-	-	-	-
Red blood cells (cells/µL)	0-2	55	-	-	-	-	-
White blood cell count (cells/µL)	0 - 5	1	-	-	-	-	-

**Figure 1:**
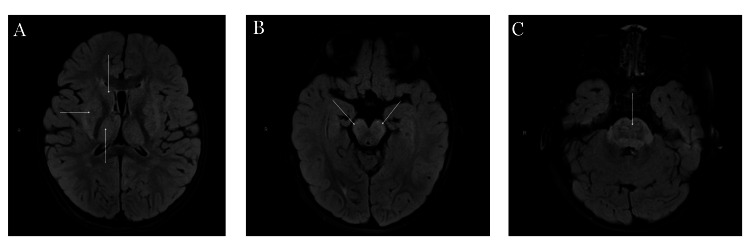
Representative axial magnetic resonance imaging (MRI) sections on fluid-attenuated inversion recovery (FLAIR). (A) Representative axial MRI sections highlight extensive signal abnormalities involving the bilateral thalami, putamen, and caudate nuclei, most conspicuous on post-contrast FLAIR imaging. (B, C) Additional extensive signal abnormality involves the brainstem, including the pons and lower midbrain, with extension into the ventrolateral medulla and the middle and superior cerebellar peduncles.

In light of reports of increasing IAE, including ANE, urgent neuroimaging was warranted to evaluate for bilateral thalamic involvement and other multifocal, symmetric gray and white matter brain lesions characteristic of this disorder. Brain magnetic resonance imaging (MRI) demonstrated widespread post-contrast fluid-attenuated inversion recovery (FLAIR) hyperintense lesions involving the basal ganglia, thalami, cortical and subcortical regions, brainstem, and cerebellar peduncles, with associated pontine hemorrhage and multifocal restricted diffusion affecting the basal ganglia, thalami, corpus callosum, cortex, hippocampi, and pons-findings consistent with the clinically suspected necrotizing encephalitis (Figure 1).

**Figure 2:**
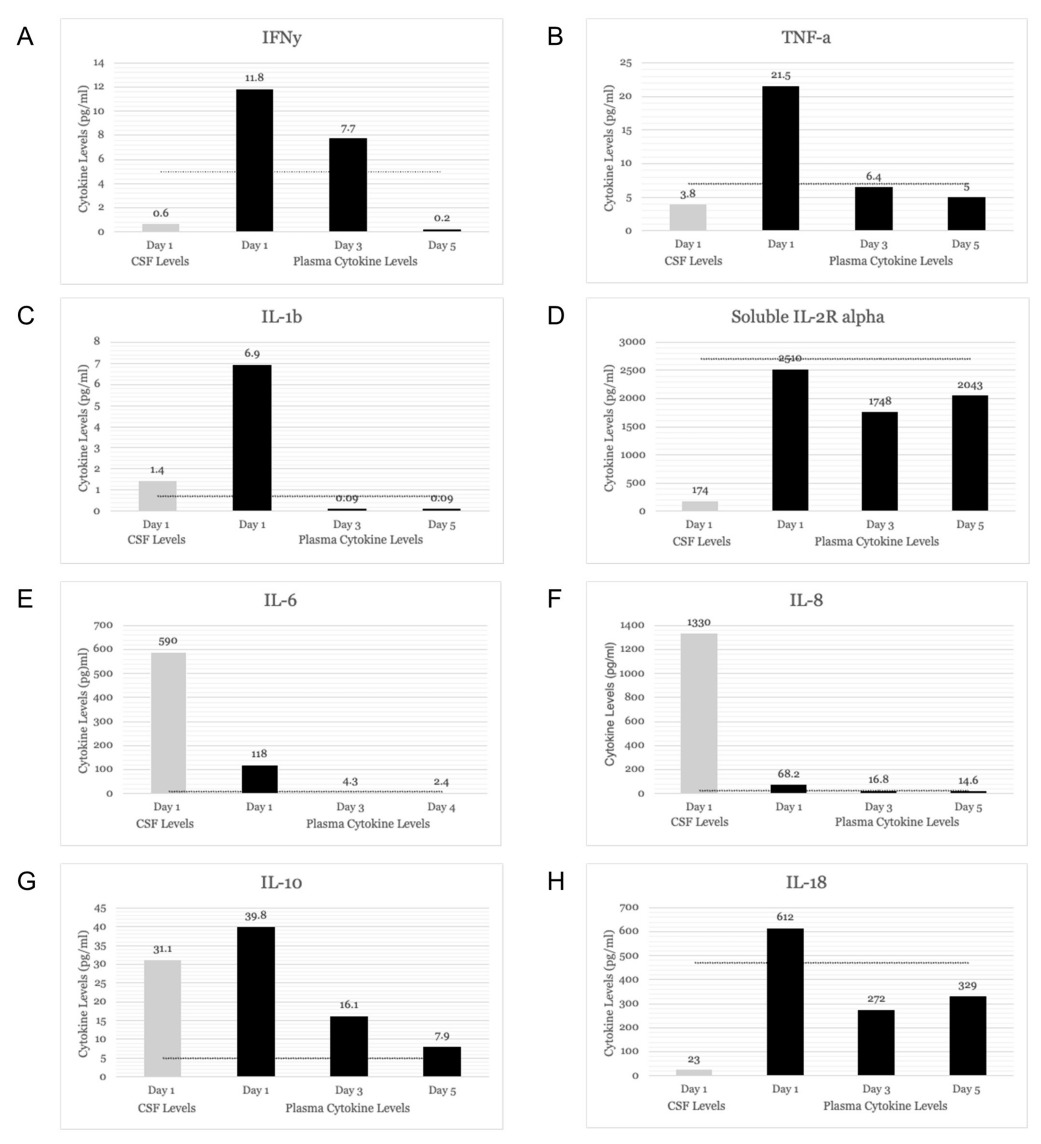
Longitudinal levels in blood and cerebrospinal fluid (CSF) Cerebrospinal fluid (gray bars) and longitudinal blood cytokine measurements (black bars) from hospital day 1 through hospital day 5. Panels show (A) interferon γ (IFNγ), (B) tumor necrosis factor α (TNFα), (C) interleukin 1 β (IL-1β), (D) soluble interleukin-2 receptor alpha (sIL-2Rα), (E) interleukin 6 (IL-6), (F) interleukin 8 (IL-8), (G) interleukin 10 (IL-10), and (H) interleukin 18 (IL-18). Blood and cerebrospinal fluid obtained on hospital day 1 were collected prior to initiation of immunomodulatory therapy. Blood obtained on hospital day 3 was collected after initiation of corticosteroids, intravenous immunoglobulin, and anakinra. Blood obtained on hospital day 5 was collected after these therapies and one session of plasma exchange. Specific cytokine values are shown above each bar. The horizontal line denotes the upper limit of normal for the blood reference range.

Because of the suspected inflammatory nature of this disease, multiple blood cytokine levels were measured (Cytokine Reference Laboratory, University of Minnesota) and found to be markedly elevated prior to immunomodulatory therapy (Figure 2). Contemporaneously collected cerebrospinal fluid demonstrated IL-6 and IL-8 concentrations exceeding those in blood, consistent with intrathecal cytokine production.

After the MRI results confirmed the diagnosis of ANE, intravenous (IV) methylprednisolone was initiated, and a total dose of 2 g/kg intravenous immunoglobulin (IVIG) was administered in divided doses over hospital days 1 and 2. There was minimal improvement after the initial therapy, so anakinra (2.5 mg/kg IV every six hours) was started on day 2 of illness. Given ongoing concern for cytokine-mediated injury and discussions with national experts and review of literature [6], plasma exchange (PLEX) was initiated on hospital day 3 as soon as appropriate vascular access could be obtained and continued for a total of five sessions.

Outcome

The patient experienced gradual improvement and was successfully extubated on hospital day 9, transferred out of the PICU on hospital day 13, and discharged to inpatient rehabilitation on hospital day 23. Rapid genome sequencing, including dedicated sequencing for mitochondrial disorders, revealed no pathogenic variants. At the time of discharge, she exhibited spontaneous movement in all extremities but with residual neurological deficits, including dysconjugate gaze and significant weakness. Seizures resolved, and levetiracetam was discontinued approximately two months after admission. At the four-month follow-up clinic visit, she met appropriate milestones, was walking and talking normally, and was discharged from all therapies, representing a full neurological recovery. Repeat MRI approximately one year after onset revealed improvement but not resolution of prior findings, with new pontine cystic changes, with a plan for routine developmental surveillance in primary care.

## Discussion

This case highlights a rare but severe neurological complication of influenza infection in a previously healthy toddler. Although influenza is often perceived to be a self-limited respiratory illness, children younger than two years are at substantially increased risk for severe disease and life-threatening complications. Current recommendations from the American Academy of Pediatrics (AAP) emphasize annual influenza vaccination beginning at six months of age [7]. In addition, prompt initiation of neuraminidase inhibitors, such as oseltamivir, is recommended for children with suspected or confirmed influenza who present with severe, complicated, or progressive illness, irrespective of vaccination status, with particular priority for children younger than two years given their vulnerability to hospitalization and death [8]. In this case, the absence of both influenza vaccination and early antiviral therapy may have contributed to the severity of the clinical course, underscoring the critical importance of prevention and timely treatment even in otherwise healthy children. Consistent with this observation, a recent pediatric ANE case series reported that 51% of affected children had no underlying medical conditions, and only 13% had received influenza vaccination, highlighting the vulnerability of previously healthy children and the ongoing need for improved vaccine uptake and early therapeutic intervention [5].

Given the potentially fulminant course of ANE, clinicians must maintain a high index of suspicion in children with influenza infection and persistent neurologic signs or symptoms lasting more than 24 hours [5]. However, the presentation necessitates careful consideration of alternative diagnoses with overlapping features, including febrile seizure, infectious meningoencephalitis, acute disseminated encephalomyelitis (ADEM), Reye syndrome, IAE, and hyperinflammatory syndromes such as hemophagocytic lymphohistiocytosis (HLH). Febrile seizures, while common in children aged 6 months to 5 years, are typically brief, self-limited, and associated with rapid recovery. By contrast, the presence of persistent seizure activity and unresponsiveness prompted neuroimaging in this case, distinguishing the presentation from an uncomplicated febrile seizure. Acute disseminated encephalomyelitis (ADEM) was considered, given its post-infectious inflammatory demyelinating nature, but its usual latency of days to weeks following infection (mean ~26 days), lower frequency of seizures at presentation, frequent CSF pleocytosis, and less symmetric neuroimaging pattern-often without prominent bilateral thalamic involvement-were discordant with this patient’s acute presentation and imaging findings [9]. Reye syndrome, historically associated with aspirin exposure during viral illness, was unlikely due to the absence of characteristic metabolic derangements, including hyperammonemia, hypoglycemia, coagulopathy, and overt hepatic failure [10]. HLH was also entertained in light of the patient’s cytokine profile; however, the lack of hallmark clinical features such as hepatosplenomegaly and cytopenias made this diagnosis less probable [11]. Among IAEs, ANE represents the most severe entity and is distinguished by rapid neurologic deterioration within days of the triggering infection, characteristic symmetric multifocal MRI lesions - most notably involving the bilateral thalami, basal ganglia, brainstem, and cerebellum - and typically minimal CSF pleocytosis with elevated protein. In this case, the abrupt onset of seizures, characteristic CSF and neuroimaging findings, and negative testing for alternative diagnoses supported a diagnosis of influenza-associated ANE.

The precise etiology and pathophysiology of ANE remain incompletely understood, but current evidence suggests that direct viral neuroinvasion is not the primary mechanism. We were not able to obtain influenza testing of the CSF to directly address this question. Alternatively, the leading hypothesis implicates a hyperinflammatory “cytokine storm” as the central driver of brain injury, whereby excessive systemic cytokine release induces neurotoxicity, alters cerebral metabolism, and disrupts the blood-brain barrier through endothelial injury [12]. These processes correlate with the characteristic findings of cerebral edema, petechial hemorrhage, and necrosis observed at autopsy in fatal cases. Typical CSF findings, namely, elevated protein in the absence of significant pleocytosis, further support a mechanism largely independent of cellular inflammation, reflecting blood-brain barrier breakdown and parenchymal injury. Although direct viral neuroinvasion is considered less likely, it remains unclear whether early antiviral therapy can attenuate cytokine responses and improve clinical outcomes by limiting early viral replication, warranting further investigation. Given the potential benefit and low perceived risk, antiviral therapy was initiated as a precaution.

Although elevated cytokines are broadly implicated in disease pathogenesis, it remains unclear which specific mediators are the primary drivers of the observed clinical manifestations and, therefore, the most relevant therapeutic targets. Comparison of cytokine levels in blood and CSF provides insight into intrathecal cytokine production, as locally produced mediators originating from microglia, astrocytes, and endothelial cells would be expected to reach higher concentrations in the CSF than in peripheral circulation. To our knowledge, this is the first report documenting a pattern of elevated CSF interleukin 6 (IL-6) and interleukin 8 (IL-8) in a case of pediatric ANE. Prior studies have demonstrated markedly elevated plasma levels of proinflammatory cytokines, including IL-6, interleukin 10 (IL-10), interleukin 1β (IL-1β), tumor necrosis factor α (TNF-α), and interferon γ (IFN-γ), similar to those observed in our patient. Among these, IL-6 and TNF-α appear particularly relevant, as high concentrations of IL-6 are neurotoxic, while TNF-α contributes to endothelial injury within the central nervous system. Our report extends the existing literature by correlating blood and CSF cytokine levels early in the disease course. Geng et al. reported elevated CSF IL-6 levels in all four pediatric ANE cases evaluated [13]. In a separate study of three adults with IAE, IL-6 and IL-8 consistently demonstrated elevated CSF-to-plasma ratios [14]. Elevated CSF concentrations of interleukin 2 (IL-2), IL-6, and IL-8 have also been reported in individual adult cases of influenza-associated ANE [15].

The triggers of the heightened inflammatory response in ANE are not fully understood, but genetic studies provide important clues. Severe influenza infection in previously healthy children and adults has increasingly been linked to monogenic inborn errors of immunity, particularly defects in innate antiviral sensing and type I and III interferon pathways. Variants in genes encoding key proteins of early viral recognition and interferon signaling (*TLR3*, *IRF7*, *IRF9*, *IFIH1/MDA5*, *GATA2*) have been shown to predispose affected individuals to life-threatening influenza pneumonitis and systemic disease [16]. Within this broader landscape of genetic susceptibility to severe influenza, a subset of patients with ANE has been linked to pathogenic variants in RAN-binding protein 2 gene (*RANBP2*) (ANE type 1), carnitine palmitoyltransferase II (*CPT II*), ribonuclease inhibitor (*RNHI*) sodium-channel alpha 1 sub-unit (*SCN1A*), and BEN-domain-containing protein (*BEND4*), and human leukocyte antigen (HLA) genes [13]. Collectively, these findings support a model in which host genetic factors, ranging from impaired antiviral defense to dysregulated inflammatory control, contribute to both severe influenza and catastrophic neurologic sequelae such as ANE, highlighting the importance of considering underlying genetic predisposition in affected patients.

Among the 37 cases of ANE reported in the recent 2024-2025 season, which included our patient, immunomodulatory therapy was given in 56%, intravenous immunoglobulin in 67%, plasma exchange in 44%, and systemic glucocorticoids in 88% [5]. Despite aggressive supportive care, outcomes were poor, mortality was 41%, and 92% of survivors had not returned to their neurological baseline at hospital discharge. Our patient presented with shock, brainstem involvement, and elevated CSF protein (>60 mg/dL), corresponding to an ANE severity score (ANE-SS) of 6 and placing her in a high-risk category for poor outcomes [17]. Given her severe disease at presentation and the historically high mortality and neurological morbidity associated with ANE, early and aggressive immunomodulatory therapy was initiated. High-dose corticosteroids and IVIG were started promptly following imaging confirmation, resulting in early improvement in systemic inflammatory markers (Figure 2). However, persistent neurological dysfunction prompted escalation of therapy. Tocilizumab, an IL-6 receptor antagonist, was considered based on published cases [18] but deferred due to concerns about minimal CNS penetration [19]. Instead, anakinra, an IL-1 receptor antagonist, was initiated based on local experience with its use in Multisystem Inflammatory Syndrome in Children (MIS-C) associated with COVID-19 and other cytokine storm syndromes, its wide therapeutic index, rapid onset of action, documented CSF penetration, and relatively low risk of secondary infection [20,21]. PLEX was pursued, guided by published literature and anecdotal reports, but its initiation was delayed due to the need for blood type matching and line placement [6]. Notably, although the patient had not returned to neurological baseline at discharge, she demonstrated continued recovery over time and ultimately returned to baseline. This favorable outcome, despite a high-risk severity score at presentation, underscores the potential impact of early and multimodal immunomodulatory therapy in modifying the course of ANE. However, our experience does not allow determination of which specific therapy, or combination thereof, was most contributory to her recovery. Furthermore, the long-term neurological improvement observed in this patient was not captured in prior case series, highlighting the importance of longitudinal follow-up to better define the natural history of ANE. Collectively, these findings emphasize the urgent need for systematic studies to evaluate individual and combination immunomodulatory treatments and establish evidence-based optimal management strategies for influenza-associated ANE.

## Conclusions

This case underscores that influenza is a systemic infection capable of causing life-threatening neurological complications, such as ANE. Early vaccination and timely antiviral therapy remain the most effective strategies to reduce disease burden in young children, particularly those under two years of age. For clinicians, maintaining a broad differential when evaluating influenza-associated neurological symptoms is essential, as rapid recognition of ANE and initiation of immunomodulatory therapy may be lifesaving. Our findings, specifically the marked CSF-predominant cytokine elevations and the favorable long-term outcome despite a high ANE severity score, highlight how individualized immunologic profiling may provide additional diagnostic and therapeutic insight. However, systematic studies are needed to define the specificity and clinical utility of cytokine measurements across overlapping inflammatory syndromes. Continued reporting of well-characterized cases will be critical to advancing understanding of ANE pathogenesis, refining treatment strategies, and improving prevention of severe neurological sequelae of influenza.
